# Sumoylation of Vimentin_354_ Is Associated with PIAS3 Inhibition of Glioma Cell Migration

**DOI:** 10.18632/oncotarget.196

**Published:** 2010-11-11

**Authors:** Liming Wang, Jian Zhang, Sipra Banerjee, Laura Barnes, Venkateswara Sajja, Yiding Liu, Baochuan Guo, Yuping Du, Mukesh K. Agarwal, David N. Wald, Qin Wang, Jinbo Yang

**Affiliations:** ^1^ School of Life Science, Lanzhou University, Lanzhou, P. R. China; ^2^ Department of Cancer Biology, Lerner Research Institute, The Cleveland Clinic Foundation, Cleveland, USA; ^3^ Tartis, Inc., Buffalo, USA; ^4^ Molecular Genetics, Lerner Research Institute, The Cleveland Clinic Foundation, Cleveland, USA; ^5^ Department of Chemistry, Cleveland State University, Cleveland, USA; ^6^ Invenio Therapeutics, Inc., Cleveland, USA; ^7^ Department of Pathology, Case Western Reserve University, Cleveland, USA

**Keywords:** SUMOylation, vimentin, PIAS3, glioblastoma cell migration

## Abstract

The invasive phenotype of glioblastoma multiforme (GBM) is a hallmark of malignant process, yet the molecular mechanisms that dictate this locally invasive behavior remain poorly understood. Over-expression of PIAS3 effectively changes cell shape and inhibits GBM cell migration. We focused on the molecular target(s) of PIAS3 stimulated sumoylation, which play an important role in the inhibition of GBM cell motility. Here we report, through the immunoprecipitation with SUMO1 antibody, followed by proteomic analysis, the identification of vimentin (vimentin_354_), a nuclear component in GBM cells, as the main target of sumoylation promoted by PIAS3.

## INTRODUCTION

Glioblastoma multiforme (GBM) represents 29% of all primary brain tumors or 5,000 new cases per year in the United States [1]. The infiltrative growth pattern of these tumors precludes curative neurosurgery and no therapeutic modality has substantially changed the outcome of patients with GBM [2]. GBM spread by active cell migration rather than by passive, hematogenous spread [3-5]. A main characteristic of GBM is the cells' extreme migration potential and topographical diffuse nature, resulting in the inability to completely dissect GBM tumors [6]. New effective therapeutic modalities for advanced and invasive GBM are desperately needed.

Protein Inhibitor of Activated STAT3 (PIAS3) was first identified as a specific inhibitor by blocking the DNA-binding activity of STAT3 and inhibiting STAT3-mediated gene activation [7]. New evidence indicates that PIAS3 suppresses STAT3-mediated signal transduction by interacting with ATBF1 [8]. PIAS3 also acts as a binding protein of other transcriptional regulators such as androgen receptor, TIF2, and NF-kappaB [9]. Recent work has revealed that PIAS3 acts as an E3-like ligase to stimulate the attachment of small ubiquitin-like modifier (SUMO) to target proteins, which act in such important cellular pathways as Wnt signaling, the p53 pathway and steroid hormone signaling [9,10].

PIAS3 induces apoptosis in prostate cancer cells both in vitro and in vivo [11]. When PIAS3 was over-expressed in GBM cells, complete suppression of growth and inhibition of cell migration was observed [12]. Considering the SUMO ligase activity of PIAS3, an obvious question is whether PIAS3-mediated sumoylation of regulators in apoptosis/migration pathways is involved in the inhibitory process. To characterize GBM's extreme migration potential, we focused on the effect of PIAS3-stimulated sumoylation on the inhibition of GBM cell migration.

## RESULTS

### Ad/PIAS3 inhibit U373 cell migration

**Figure 1:**
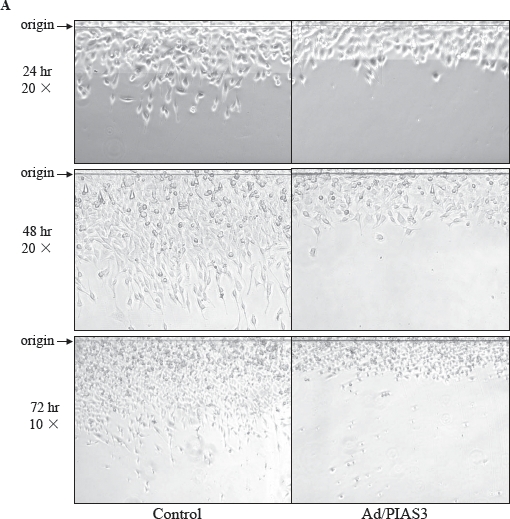

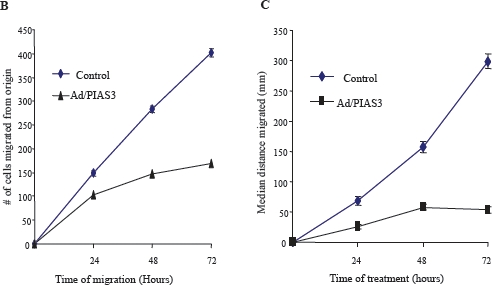

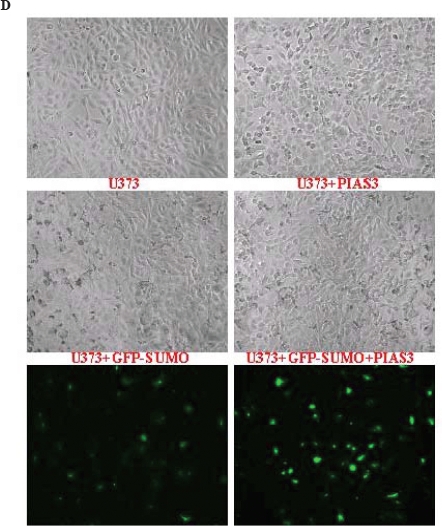
Inhibition of U373 cell migration by Ad/PIAS3 **A.** Confluent U373 cells were cut and lifted at the origin (arrow), and infected with 5 pfu/cell Ad/PIAS3. Images (original magnification × 10 or × 20) of migration were taken by phase contrast microscopy at the times indicated. Sizes of images are: 420 × 210 nm for 24 hrs, 420 × 315 nm for 48 hrs, 840 × 630 nm for 72 hrs. **B.** Number of cells migrated out from the origin; **C.** Median distances of cells migrated from the origin. Ad/PIAS3 infected cells showed a decreased cell number and shorter distance in migration compared with control cells (mean of three independent experiments, Student's t-test P<0.01). **D.** PIAS3 promotes sumoylation in U373 cells. Phase-contrast microscopic images collected 24 hours of control (U373), Ad/PIAS3 infected (+ PIAS3), EGFP-SUMO1 transiently transfected (+ GFP-SUMO), and Ad/PIAS3 infected U373 cells after EGFP-SUMO1 transfection (+ GFP-SUMO + PIAS3). Fluorescent EGFP-SUMO1 clearly demonstrated that PIAS3 increased SUMO1 expression and accumulation of EGFP-SUMO fusion protein in nuclei (lower 2 panels, respectively).

When quiescent GBM U373 cells were infected with a low titer (5 pfu/cell) of Ad/PIAS3, the cell's morphology was changed, and cells became rounded and lost processes (Figure 1A). As demonstrated in the “artificial wound assay”, a two-dimensional cell motility system [13], U373 cell migration was clearly inhibited compared with the cells infected by Ad/EGFP (Figure 1A). The inhibition of motility can be reflected by decreased cell number (Figure 1B) and reduced distance (F polymerization at 72OC for igure 1C) migrated out from the origin of artificial wounding. The control cells have long processes toward the direction of movement. In contrast, Ad/PIAS-treated cells form few, if any, long processes, and such processes do not extend in forward direction.

To examine whether PIAS3 promotes sumoylation, U373 cells were transfected with enhanced green fluorescent protein (EGFP)-tagged SUMO1 before infection. As shown in Figure 1D, PIAS3 did promote accumulation of EGFP-SUMO1 fusion proteins in the nuclei, reflected by increased intensity of green fluorescence in Ad/PIAS3-treated GBM cells.

### PIAS3 promotes Vimentin354 sumoylation

**Figure 2:**
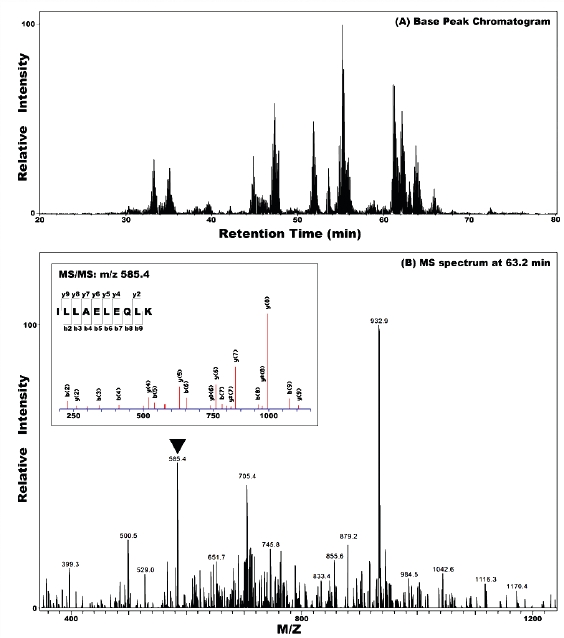

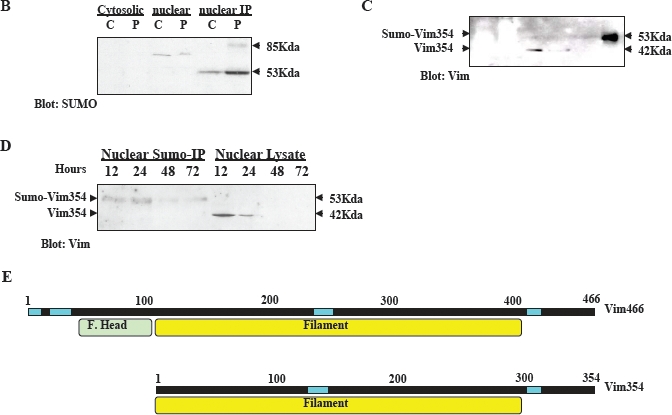
Validation of PIAS3 stimulated Vimentin_354_ sumoylation in the nucleus of U373 cell **A.** Representative Nano-HPLC/MS/MS analysis for protein identification. The complete vimentine_354_ peptide sequence is given on the top panel. Middle panel: Nano-HPLC/MS/MS base peak chromatogram of the tryptic digests of gel slice in the mass range of 53 kDa; lower panel: MS spectrum at retention time of 63.2 min, and MS/MS spectrum of peptide ILLAELEQLK unique to the Vimentin_354_ protein. **B & C.** Quiescent cells were infected with Ad/PIAS3 for 24 hrs and cytosolic or nuclear protein lysate was prepared. Then 80% of the nuclear protein immunoprecipitated by SUMO antibody (nuclear IP) from Ad/EGFP mock control (C) or Ad/PIAS3 (P) infected U373 cells were immunoblotted to demonstrate the same motility of both SUMO (**B**) and vimentin_354_ (**C**). Fifty microgram each of cytosolic and nuclear proteins was blotted to display the molecular weight of unmodified vimentin_354_ at about 41.5 kDa. **D**. Sumoylation of nuclear vimentin_354_ at different time points after Ad/PIAS3 infection. **E**. Comparison of vimentin_354_ and vimentin_466_. With 91% of homology, each has a filament rod domain, essential for protein-protein interaction. vimentin_354_ lacks an IF head domain, which is responsible for DNA binding, at the N-terminus.

The next question was what are the molecular targets of PIAS3-stimulated sumoylation, which play an important role in the inhibition of GBM cell motility. The PIAS3 had been mainly localized in nuclei [9,12] and fluorescent EGFP-SUMO1 was accumulated in the nucleus upon PIAS3 stimulation. Unlike most sumoylated proteins identified by over-expressed tag-SUMO, our goal was to check the stimulatory effect of PIAS3 on endogenous SUMO. Then, agarose conjugated SUMO1 antibody (Santa Cruz) was used for immunoprecipitation (IP) of nuclear proteins. As shown in Figure 2B, the two most prominent proteins, with molecular weights of about 53 kDa and 86 kDa, can be identified by SUMO antibody in the Ad/PIAS3-infected cells, indicating that PIAS3 indeed promotes endogenous sumoylation of nuclear proteins. Because of the limited resolution of western blot results, we could not detect the presence of more sumoylated but less-abundant proteins by SUMO-IP.

Coomassie Blue-stained nuclear IP protein bands separated on polyacrylamide gel were excised, and protein in gel or the whole IP product on agarose beads was trypsinized. Then digested peptides were analyzed by high performance liquid chromatography and mass spectrometry/mass spectrometry (HPLC-MS/MS). Surprisingly, the vimentin_354_ (Accession No. AAA61281) [14] was identified in all four independent SUMO-IP followed by MS-MS analysis from two MS cores (Figure 2A). The molecular weight of vimentin_354_ is about 41.5 kDa, when modified by SUMO1 (MW: 11 kDa), should move to 52.6 kDa. Western blot with goat anti-vimentin antibody confirmed the sumoylation of vimentin_354_, which was only found in the IP product of nuclear lysates (Figure 2B and C). Vimentin_466_ (Accession No. NP_003371, MW 53.6 kDa) was also identified from the band at about 86 kDa, but failed to be recognized by goat anti-vimentin antibody. When the SUMO-IP pulled nuclear proteins from different time points were analyzed by western blot, vimentin_354_ was found to decrease with time of Ad/PIAS3 infection, reducing to undetectable level after 48 hours (Figure 2D).

## DISSCUSSION

Vimentin has been used as a molecular marker for GBM and astrocytomas [15-17]. Gene expression profiles assessed from laser capture-micro-dissected GBM cells have shown high levels of vimentin in the tumor core [18]. Theoretical analysis predicted two lysines with high probability (K261 and K201), and one lysine with low probability (K111) [19] as the putative SUMO site. Calculated from the molecular weight on western blot, only one SUMO was attached to vimentin_354_, result in the major band on SUMO-IP expected at 53 kDa. Sub-cellular localization of vimentin_354_ was predicted to be in the nucleus with 76.7% of reliability by theoretical calculation [20], which matched our observation. Compared with the abundant vimentin_466_ located in both nucleus and cytoplasm, the nuclear vimentin_354_ was expressed at very low level, therefore, vimentin_354_ could not be visualized by fluorescence immunohistochemistry (data not shown). The absence of lysine or arginine residues in the C-terminus of mammalian SUMO makes it difficult to map sumoylation sites [21], which may explain our failure to identify SUMO peptide in our MS/MS analysis.

Vimentin, as a primordial component of the cytoskeleton and the nuclear envelope, belongs to the class-III intermediate filaments (IF) found in mesenchymal cells. The de novo expression of vimentin is frequently involved in the epithelial-to-mesenchymal transition (EMT) associated with increased invasive/migratory properties of epithelial and cancer cells [22]. In prostate cancer cells, the level of vimentin expression has been correlated with cell motility, and the majority of poorly differentiated cancers and bone metastases showed high vimentin expression in tumor cells [23]. When vimentin was experimentally reduced, the cell's invasive potential could be effectively abolished in the *in vitro* Matrigel invasion assay [24]. Over-expression of vimentin in breast cancer model leads to augmentation of motility and invasiveness in vitro, which can be transiently down-regulated by antisense oligonucleotides to vimentin [25]. Using an in vitro wound-healing model, it had been demonstrated that vimentin is transiently associated with, and could be functionally involved in the migratory status of breast epithelial cells [26]. Our observation hinted that sumoylation can be another way of removing functional vimentin, leading to the inhibition of GBM cell motility.

Compared with vimentin_466_, vimentin_354_ lacks 112 residues at the N-terminus, representing the IF head (DNA binding) sequence, which is able to alter nuclear architecture and chromatin distribution. With 91% homology to vimentin_466_ (Figure 2E), vimentin_354_ has the same B-Box-type zinc finger, zinc-binding domain, RING-finger (Really Interesting New Gene) domain, involved in mediating protein-protein interactions, which have a wide range of functions such as signal transduction and development. Migration is closely linked to cell shape and to mechanics of the cytoskeleton. Other than the current model of viscous fluid-like cytoplasm and elastic membrane to be the major load-bearing elements, the idea that “hard-wired” transmembrane receptors, cytoskeleton filaments (including vimentin), and nuclear scaffolds are responsible for cell shape control had been proposed [27]. Vimentin_354_ may serve as the end connection molecule in the nucleus to hold the entire IF network, which is crucial for supporting the three-dimensional shape of the cell skeleton.

Post-translational modification by SUMO-1 is a highly conserved process in eukaryotic cells and plays important regulatory roles in many cellular processes. Since vimentin_354_ is the major target of endogenous sumoylation in the nucleus upon PIAS3 stimulation, it may play a key role in maintaining the normal cell shape and motility of GBM cells. Targeting the cell motility component actin had been proposed to combat cancer cell migration [28]. Our data suggest that vimentin_354_ may be added to the therapeutic target for fighting GBM.

## MATERIALS AND METHODS

### Cell culture and Ad/PIAS3 infection

Glioblastoma U373 cells were cultured in Dulbecco's modified Eagles medium (DMEM) containing 5% fetal bovine serum and antibiotics. Cells were incubated at 37C in a humidified atmosphere containing 5% CO_2_. Adenovirus5 with PIAS3 was constructed. The entire coding sequence of human PIAS3 (Genbank accession number NM_006099) was amplified by RT-PCR, cloned into the pShuttle-CMV vector, and sent to Cleveland Clinic Foundation Virus Core for adenovirus construction and purification. Infection was performed by diluting the virus to the appropriate concentration in medium. Adenovirus5 vector was used as control.

### Cell count

50,000 U373 cells were seeded in each well of 12-well tissue culture plate, and infected with 100 pfu/cell Ad/PIAS3. Cells were trypsinized at different time points, and counted as total cell number or cell survival by trypan blue exclusion. The numbers of average and standard error are obtained from triplet wells.

### In-gel digestion of SDS-PAGE separated and coomassie-stained gel

Coomassie-stained polyacrylamide gel containing protein bands of interest were excised. gels were destained with 50% acetonitrile in 100 mM ammonium bicarbonate, and then 100% acetonitrile. The proteins were reduced in the gels with 20 mM dithiothreitol (DTT) at room temperature for 30 min, followed by alkylation with 50 mM iodoacetamide in 100 mM ammonium bicarbonate for 30 min in the dark. After treatment, the reagents were removed and the gel pieces were washed with 100 mM ammonium bicarbonate and then dehydrated in acetonitrile. The dried gel pieces were then rehydrated in a solution of sequencing grade, modified trypsin in 50 mM ammonium bicarbonate for digestion overnight. Tryptic peptides were extracted from the gel with 50% acetonitrile in 5% formic acid. The LC-MS/MS analysis is performed on Bruker HCT 3000 plus ESI-IonTrap Mass Spectrometer coupled with Agilent 1100 HPLC system. An additional Shimadzu LC-10ADvp HPLC pump is used for sample loading. Tryptically digested sample is injected and first loaded onto a Rheodyne Peptide Captrap (Michrom BioResources, Inc.), then eluted through a capillary RP-HPLC column (10 cm long, 300 μm ID, 5Å C18, from Grace Vydac). The elution gradient is: 2% B for 10 min; 5%-45% B for 200 min; 45%-85% B for 30min; 85% B for 30 min. The ion-trap mass spectrometer is set in auto ms/ms mode; number of precursor ions is 3. The collected MS/MS data are processed (compound finding and deconvolution) by Bruker Data Analysis, version 3.1.

### Immunohistochemistry

Slides of human normal organs and human various tumors were purchased from Imgenex. The paraffin was removed by xylene and followed by sequencial ethanol wash. Antigens were retrieved by boiling the slides for 5 minutes in 10 mM Sodium Nitrate (pH 6.0) and rehydrated in PBS. The slides were first blotted in PBS with 0.5% BSA and 0.2% cold water fish gelatin (PBG), then incubated in PBG with anti PIAS3 antibody. Biotinylated secondary antibody and Streptoavidin conjugated with horseradish peroxidase (ABC kit, Vector Labs) were applied to the slides, color develop was achieved by using DAB kit (Vector Labs) following the manufacturer's instructions. All images were captured at the same light intensity, brightness and contrast.

### Human brain tumor RNA

All original brain tumors were obtained from the Department of Neurosurgery, The Cleveland Clinic Foundation, and diagnosed as glioblastoma, astrocytoma and oligodendrocytoma by surgical pathology Pieces of fresh brain tumors were fast frozen in liquid nitrogen, and ground into fine powder for RNA isolation.

### Western blot

The U373 cells were lysed in 1% Triton X-100 lysis buffer containing protease inhibitors. Cell debris was removed by centrifugation. 20 mg of protein from each sample was separated on SDS-PAGE gel, transferred to PVDF membrane, and blotted with anti PARP antibody. Positive bands on the blots were detected by using ECL-Plus kit (Amersham-Pharmacia) following manufacturer's instruction.

### RT-PCR amplification of PIAS3 mRNA sequences

Total RNA extracted from normal white matter and brain tumors was reverse transcribed to cDNA. PIAS3's coding sequences from nucleotide 1 to 861 was amplified by 35 cycles of denaturing at 95°C for 1', annealing at 55°C for 1', and polymerization at 72°C for 1' 30”. A 516 bp β-actin fragment was also amplified for quality control.

### Measurement of glioma cell motility

Cell migration was measured by the ‘artificial wound’ method as described [8]. Confluent U373 cells in 12-well tissue culture plates were cut-lift (wound) with a razor and 50 pfu/cell Ad/PIAS3 was added to the medium for infection. Control cells were infected with Ad/GFP at the same concentration. A digital camera on phase-contrast microscope was used to capture images of cell migration. In each well, three random fields were chosen to count the number of cells that crossed the origin line and to calculate the median distance the cells migrated. The data from triplicate wells were expressed as the mean and standard error of the number of migrating cells, as well as the median distance. All experiments were done at least three times.
